# Soluble Cytokine Receptors (sIL-2Rα, sIL-2Rβ) Induce Subunit-Specific Behavioral Responses and Accumulate in the Cerebral Cortex and Basal Forebrain

**DOI:** 10.1371/journal.pone.0036316

**Published:** 2012-04-27

**Authors:** Steven S. Zalcman, Ankur Patel, Ruchika Mohla, Youhua Zhu, Allan Siegel

**Affiliations:** Department of Psychiatry, UMDNJ-New Jersey Medical School, Newark, New Jersey, United States of America; Zhejiang University School of Medicine, China

## Abstract

Soluble cytokine receptors are normal constituents of body fluids that regulate peripheral cytokine and lymphoid activity. Levels of soluble IL-2 receptors (sIL-2R) are elevated in psychiatric disorders linked with autoimmune processes, including ones in which repetitive stereotypic behaviors and motor disturbances are present. However, there is no evidence that sIL-2Rs (or any peripheral soluble receptor) induce such behavioral changes, or that they localize in relevant brain regions. Here, we determined in male Balb/c mice the effects of single peripheral injections of sIL-2Rα or sIL-2Rβ (0–2 µg/male Balb/c mouse; s.c.) on novelty-induced ambulatory activity and stereotypic motor behaviors. We discovered that sIL-2Rα increased the incidence of *in-place* stereotypic motor behaviors, including head up head bobbing, rearing/sniffing, turning, and grooming behavior. A wider spectrum of behavioral changes was evident in sIL-2Rβ-treated mice, including increases in vertical and horizontal ambulatory activity and stereotypic motor movements. To our knowledge, this is the first demonstration that soluble receptors induce such behavioral disturbances. In contrast, soluble IL-1 Type-1 receptors (0–4 µg, s.c.) didn't appreciably affect these behaviors. We further demonstrated that sIL-2Rα and sIL-2Rβ induced marked increases in c-Fos in caudate-putamen, nucleus accumbens and prefrontal cortex. Anatomical specificity was supported by the presence of increased activity in lateral caudate in sIL-2Rα treated mice, while sIL-2Rβ treated mice induced greater c-Fos activity in prepyriform cortex. Moreover, injected sIL-2Rs were widely distributed in regions that showed increased c-Fos expression. Thus, sIL-2Rα and sIL-2Rβ induce marked subunit- and soluble cytokine receptor-specific behavioral disturbances, which included increases in the expression of ambulatory activity and stereotypic motor behaviors, while inducing increased neuronal activity localized to cortex and striatum. These findings suggest that sIL-2Rs act as novel immune-to- brain messengers and raise the possibility that they contribute to the disease process in psychiatric disorders in which marked increases in these receptors have been reported.

## Introduction

Soluble cytokine receptors (SCR) are circulating proteins that lack the extracellular portion of their membrane counterparts. SCRs are normal constituents of body fluids that regulate cytokine and lymphoid activity [1]–[3]. The first SCR to be characterized - the soluble interleukin-2 receptor (sIL-2R), regulates IL-2 family cytokine activity [1]. The membrane bound IL-2 receptor is composed of α, β, and γ subunits, and is normally expressed on T and B lymphocytes, among other cells in the immune and central nervous systems [4], [5]. While the bulk of data concerning soluble forms of the IL-2R has focused on the alpha subunit (sIL-2Rα), a soluble form of the beta subunit (sIL-2Rβ) has also been characterized [6].

Serum levels of sIL-2Rs are markedly increased in disease states associated with immune activation, such as autoimmune, infectious, and neoplastic disorders [7], [8]. A positive correlation between sIL-2R's levels and disease progression has been observed in certain immunologically based disorders [9]. As such, they may serve as important biomarkers of disease activity in conditions linked to immune activation. An intriguing aspect to the sIL-2R-immune activation relationship is that increased levels of sIL-2Rs are evident in psychiatric disorders linked to autoimmune conditions and associated with a psychomotor activation, repetitive stereotyped movements, and other motor disturbances. For example, numerous investigators have shown that serum and/or cerebrospinal levels of sIL-2Rs are increased in schizophrenic patients [10]–[15]. It was further shown that increased levels of sIL-2Rα occur coincident with an increased expression of motor disturbances in subsets of schizophrenic patients, including neuroleptic-naïve patients [16] and medicated patients with tardive dyskinesia [17]. sIL-2Rα are also increased in acute mania but not during remission [18], [19], suggesting a link between sIL-2R and psychomotor activation.

In view of the link between sIL-2Rs and psychiatric disorders involving motor disturbances, an intriguing possibility is that sIL-2Rs may precipitate such disturbances. The fact that sIL-2Rs regulate cytokine and lymphoid activity supports this view. However, there is no evidence that sIL-2Rs (or any soluble receptor) provoke such behavioral changes. As well, there is no evidence that peripheral sIL-2Rs accumulate in the brain. Such a finding would have wide ranging implications. Accordingly, in the present investigation we sought to determine in male Balb/c mice whether single peripheral injections of sIL-2Rα or sIL-2Rβ induce variations in motor activity and repetitive stereotyped movements, and whether they stimulate activity and accumulate in brain regions that act as important neural substrates of such behaviors, including the caudate-putamen, nucleus accumbens, and motor and prefrontal cortices.

## Materials and Methods

### Subjects

All methods and procedures were approved by Institutional Animal Care and Use Committee (IACUC) of UMDNJ, Newark, NJ. A total of 92 adult male Balb/c mice (Charles River Laboratories, Wilmington, MA) were used in the present study. We used male Balb/c mice to maintain consistency with previous studies from our laboratory examining behavioral and neural consequences of IL-2 treatment [20], [21]. The mice were housed in standard polypropylene ‘shoebox’ cages in groups of four until testing whereupon they were individually housed. The animals were maintained on a 12 hour light/12 hour dark light cycle (7 am–7 pm), and permitted free access to standard laboratory chow and water. For behavioral testing, 4–7 mice/group were used for the tests and received single subcutaneous injection of either Saline, sIL-2Rα (0.5 µg–2 µg/mouse), sIL-2Rβ (1 µg–2 µg/mouse) or sIL-1R1 (1 µg–8 µg/mouse). For fluorescent labeling we used 4 mice in each group, whereas 8–12 mice/group were used for c-fos immunohistochemistry experiment. Behavioral experiments were conducted multiple time using different mice in order to determine stable and reproducible effects of drug administration.

### Drugs

Recombinant human sIL-2Rα was obtained from Peprotech (Rockhilll, NJ), where as recombinant human carrier free sIL-2Rβ and sIL-1R1 were obtained from R&D Systems (Minneapolis, MN).

### Behavioral Testing

Immediately following injections, the mice were individually placed into a test arena (TruScan Behavioral Monitoring System; Coulbourn Instruments, PA) in normal illumination for 2-hr. A series of behavioral measurements were recorded including locomotion, vertical activity, horizontal and vertical stereotypic movements, jumps, and turns. *Locomotion* was defined as the sum of all vectored coordinate changes in the floor plane, and included floor plane total movement distance less the stereotypic movement distance. *Vertical activity* was defined as total movements in the vertical plane. Each movement is a series of successive coordinate changes with no rest (same coordinates) for at least 1 sample interval. *Horizontal stereotypic movements* were defined as the total number of coordinate changes ±1.499 beam spaces in the floor plane (X and Y) dimension and back to the original point that do not exceed 2 seconds apart. Three such movements must be made before a stereotypy episode starts. When it does, the qualifying 3 movements are included in the total number of moves. *Vertical stereotypic movements'* measures are analogous to those in the floor plan except that only vertical movements are measured. *Jumps* were defined as the total number of time-contiguous 0-0 coordinate sets that do not exceed 2 seconds. *Turns* were defined as movements where the animal enters 4 radially-contiguous quadrants in ascending or descending order, without interruption.

The test sessions were also filmed with a VHS camera, and at a later date, an experienced rater blinded to the treatment groups scored the incidence and duration of stereotypic behaviors, which included sniffing, head bobbing, and intense grooming. We selected these stereotypies based on our findings that IL-2 treatment induces an increase in stereotypic motor behavior [21], unpublished data showing that such behaviors may be induced by elements of an activated immune system, and their utility as animal analogous of repetitive stereotyped movements. The time engaged in *head bobbing* was defined as the time (sec) engaged in repeated vertical head bobbing. A minimum of 3-sec of uninterrupted head bobbing was defined as one episode. The duration of such episodes was recorded with a stopwatch. *Sniffing* was defined as the time (sec) engaged in head-up and head-down sniffing with the head/snout directed towards the top of the cage or down toward the floor of the cage, respectively. We also measured the duration of such episodes as well as continuous sniffing for at least 5-sec. Measurements were taken 20-, 40-, 60-, 80-, 100- and 120- min after injection in 2-min epochs. Results are presented as totals (± SEM) for the entire session.

### c-Fos immunohistochemistry

The mice received a single injection of saline, sIL-2Rα (1 µg/mouse, s.c.) and sIL-2Rβ (2 µg/mouse, s.c.) and immediately thereafter were placed into an open field for 2-hr. Immediately following the test session, the mice were deeply anesthetized with Sodium pentobarbital (60–80 mg/kg, i.p.) and transcardially perfused with 0.9% saline (pH 7.2) followed by 4% paraformaldehyde (pH 7.4). After perfusion, brains were removed from the skull and then placed in 4% paraformaldehyde solution at 4°C for overnight. The brains then were transferred to 30% sucrose solution at 4°C until they sank to the bottom whereupon they were frozen in a cryostat (Leica CM1900) at −20°C for slow freezing. Sections were cut at 30 µm thickness and alternate sections were transferred to 24 well plates containing PBS, and washed for 30-min. Sections were then incubated on a rocker table with rabbit antibody to c-fos protein (Santa Cruz, CA) diluted in 1∶500 in a PBS solution containing 1% normal goat serum and 0.3% Triton X-100 overnight at 4°C. The next day, sections were washed with PBS and incubated in biotinylated goat anti-rabbit antibody (1∶333, Vector Labs, Burlingame, CA) in PBS for 1 h. Sections were then rinsed in PBS, incubated for 90- min in Avidin–Biotin/Peroxidase (ABC Elite, Vector Labs), and washed 3 times in PBS. The sections were then treated with a ready-made stabilized solution of active Diaminobenzidine (‘Stable DAB’, Invitrogen) until completion of the reaction (10 min), and again washed, then dehydrated with alcohol, treated with Xylene, and cover slipped with Permount (Sigma-Aldrich). Slides were viewed with an Olympus AX-70 microscope and photographed with a Magnafire digital camera. Composite images were taken at 4× magnification using Neurolucida software (Microbrightfield Inc., Williston, VT).

### Fluorescent labeling for sIL-2Rα and sIL-2Rβ

Brain sections were first blocked with 5% normal goat serum containing 0.3% Triton X-100 in PBS for 1 hr and then incubated with anti-human sIL-2Rα monoclonal antibody (1∶100 dilution) or anti-human IL-2Rβ affinity purified polyclonal antibody (1∶100 dilution) overnight at 4°C followed by incubation with goat anti-human IgG-FITC (green, 1∶200; Santa Cruz, CA) secondary antibody for 1 h at room temperature. Then the slices were mounted on a slide, and viewed under an Olympus fluorescence microscope.

### Image Analysis

We defined the areas to be counted bilaterally as: (1) (a) Caudate-Putamen, and (b) sub-territories within the caudate-putamen, including the dorsomedial (DM), dorsolateral (DL), central (CT), ventromedial (VM), and ventrolateral (VL) aspects; (2) (a) Nucleus Accumbens, and (b) compartments within the nucleus accumbens, including the shell (SH) and core (CO); (3) Motor Cortex, including M1 and M2 divisions; (4) Cingulate Cortex; (5) Infralimbic Cortex; (6) Sensorimotor cortex; and (7) Prepyriform cortex. Black and white Kodalith image masks were subsequently created, permitting Fos-immunoreactive cells to be converted into individual black dots, while everything else was eliminated as a white background. These image masks were then processed and particles no smaller than 10 pixels (to exclude background staining artifacts), and no larger than 50 pixels (to eliminate large areas of immunoreactivity such as non-specific staining at the edge or at folds in the tissue were counted. Each Fos-positive nucleus was marked with an asterisk. Using Neuroexplorer (Microbrightfield Inc., Williston, VT) software, we then counted the number of immunoreactive nuclei present within each of the pre-defined areas of different brain regions. Data from c-Fos immunohistochemistry (number of Fos-immunoreactive cells) were counted from 3 sections per animal and 4 animals for each treatment were then submitted to one-way ANOVA, with an accepted level of significance of p<0.05.

We used Adobe Photoshop to generate an overlay image for c-Fos and Immunofluorescent combined image. Individual images of c-Fos and Fluorescent staining of each region from various treatment groups were selected and overlayed to visualize both staining at optimal level.

## Results

### Behavior of sIL-2Rα treated mice

**Figure 1:**
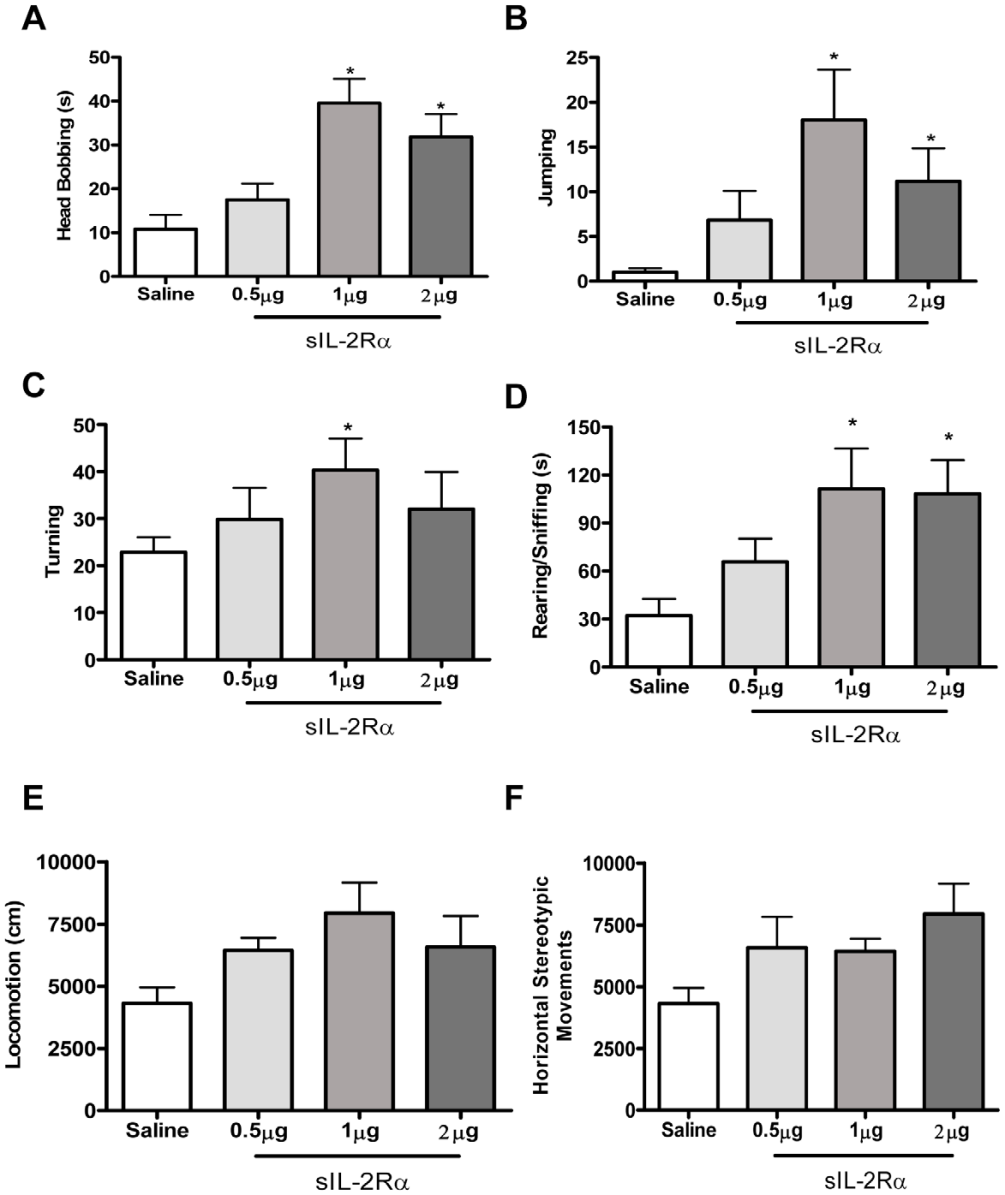
Behavioral effects induced by single injection of sIL-2Rα. sIL-2Rα –induced locomotor changes and repetitive stereotyped movements. Mean (± S.E.M.) activity scores for; (A) Head bobbing (B) Jumping (C) Turning (D) Rearing/Sniffing duration (E) Locomotion and (F) Horizontal Stereotypic Movements following single injections of sIL-2Rα (0, 0.5,1 and 2 µg/mouse, s.c.).

sIL-2Rα treated mice displayed increased novelty-induced stereotypic behaviors. In particular, sIL-2Rα treated mice showed dose-dependent increased head-bobbing [F (3, 19) = 7.62, p = 0.002; figure 1a], Jumping [F (3, 20) = 2.83, p = 0.03; figure 1b], and turning (F (3, 20) = 1.87, p = 0.04) (figure 1c). The sIL-2Rα treated mice also spent more time exhibiting in-place stereotypic activity as evident by increased rearing (F (3, 19) = 3.79, p = 0.01) (figure 1d). Additionally, these mice showed a tendency for higher locomotion (F (3, 20) = 3.18, p = 0.06) (Figure 1e), with more horizontal stereotypic movement as compared to control mice; however, these findings were not statistically significant (F (3, 20) = 4.23, p = 0.07) (figure 1f).

### Behavior of sIL-2Rβ treated mice

**Figure 2:**
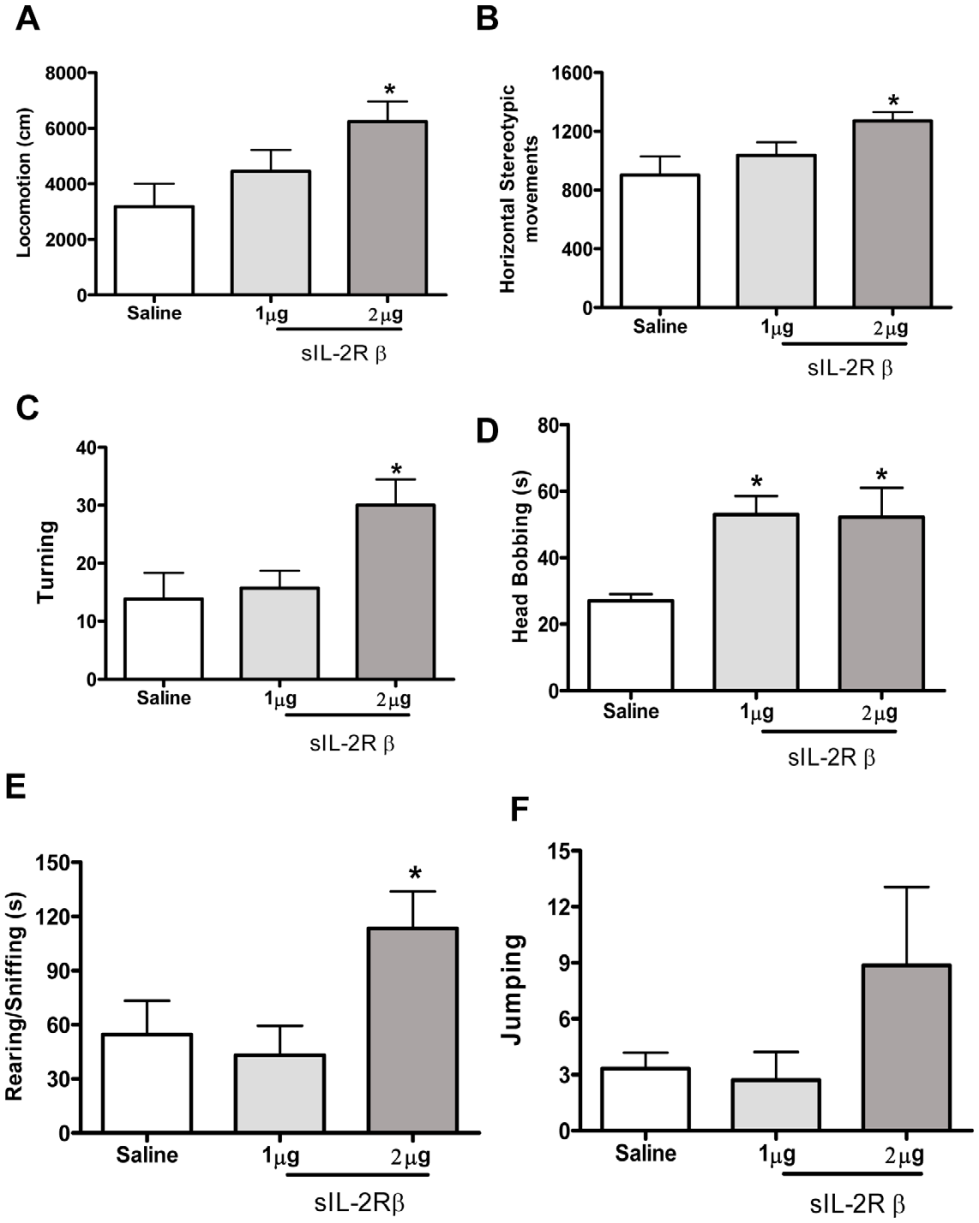
Behavioral effects induced by single injection of sIL-2Rβ. sIL-2Rβ–induced locomotor changes and repetitive stereotyped movements. Mean (± S.E.M.) activity scores for; (A) Locomotion (B) Horizontal Stereotypic Movements (C) Turning (D) Head bobbing (E) Rearing/Sniffing duration and (F) Jumping following single injections of sIL-2Rb (0, 1 and 2 µg/mouse, s.c.).

sIL-2Rβ treated mice displayed increased novelty-induced locomotion and stereotypic behaviors as compared to control mice. sIL-2Rβ treated mice showed dose-dependent increased locomotion (F (2, 17) = 3.85, p = 0.04) (Figure 2a), increased horizontal stereotypic movement (F (2, 17) = 3.95, p = 0.04) (figure 2b) and turning (F (2, 17) = 3.20, p = 0.05) (figure 2c). The sIL-2Rβ treated mice spent more time exhibiting in-place stereotypic activity as evident by increased head-bobbing [F (2, 17) = 6.02, p = 0.02; figure 2d] and also increased rearing (F (2, 17) = 3.72, p = 0.01) (figure 2e). These mice also showed a tendency for more Jumping compared to controls, but this finding was not statistically significant [F (2, 17) = 1.56, p = 0.24; figure 2f].

### Behavior of IL-1R1 treated mice

As shown in Table 1, when treated with sIL-1R1 mice were not significantly different from controls (saline treated mice) for locomotor activity (p = 0.15), stereotypic movements (p = 0.5), turning (p = 0.47), rearing (p = 0.94) and head bobbing (p = 0.37). These findings demonstrate that the specificity of stereotypic effects described above for sIL-2Rs do not extend to animals treated with sIL-1R1.

**Table 1:** Effect of various doses of sIL-1R1 on different open field behaviors.

Parameter	Saline	sIL-1R1 µg	sIL-1R 2 µg	sIL-1R 4 µg	sIL-1R 8 µg	f	p
Locomotion (cm)	6624.08±3198.82	7762.83±3394.46	7518.11±3540.1	7393.47±1909.66	4499.7±2113.78	f (4, 34) = 1.83	0.15
Rearing Episodes (#)	40.3±21.9	44.43±18.4	37.43±12.4	37.7±18.24	36.2±26.62	f (4, 34) = 0.19	0.94
Turning	29.5±13.91	33±11.87	35±17.37	30.88±10.04	21.66±13.93	f (4, 34) = 0.91	0.47
Sniffing (sec)	461.5±135.41	389.6±77	415.75±91.14	455±107.53	N/A	f (3,12) = 0.46	0.71
Head Bobbing	36.33±3.78	37±17.8	14.2±19	32±31.66	3±4.24	f(3,14) = 1.12	0.37
Vertical Stereotypy Moves	36.25±37.03	36±24.74	15.5±3.31	42.67±23.11	N/A	f (3,12) = 0.82	0.5
Vertical Stereotypy Episodes	16.25±16.04	15.8±10.23	7±1.63	18.67±9.01	N/A	f (3,12) = 0.87	0.48

Table shows effects of various doses of single injection of sIL-1R1 on various behaviors in open field testing. Effects was considered significance if p<0.05.

### Effects of single injections of recombinant sIL-2α and sIL-2Rβ on c-Fos immunoreactivity in cortico-striatal regions

We showed that single injections of sIL-2Rα and sIL-2Rβ induce subunit-specific alterations in novelty stress-induced motor activity, which includes increased locomotion, vertical activity, and stereotypic behaviors. These findings imply that these soluble receptors modulate cell activity in cortico-striatal regions. However, to our knowledge, there is no evidence that a soluble receptor of any kind modulates activity in these (or other) brain regions. Thus, we sought to determine whether sIL-2Rα and sIL-2Rβ increase c-Fos expression in subregions of the caudate-putamen, nucleus accumbens, and motor cortex. In order to further examine stereotypic responses, we extended our analysis to sniffing behavior as it may relate to activation of the prepyriform cortex in response to the presence of odors.

**Figure 3:**
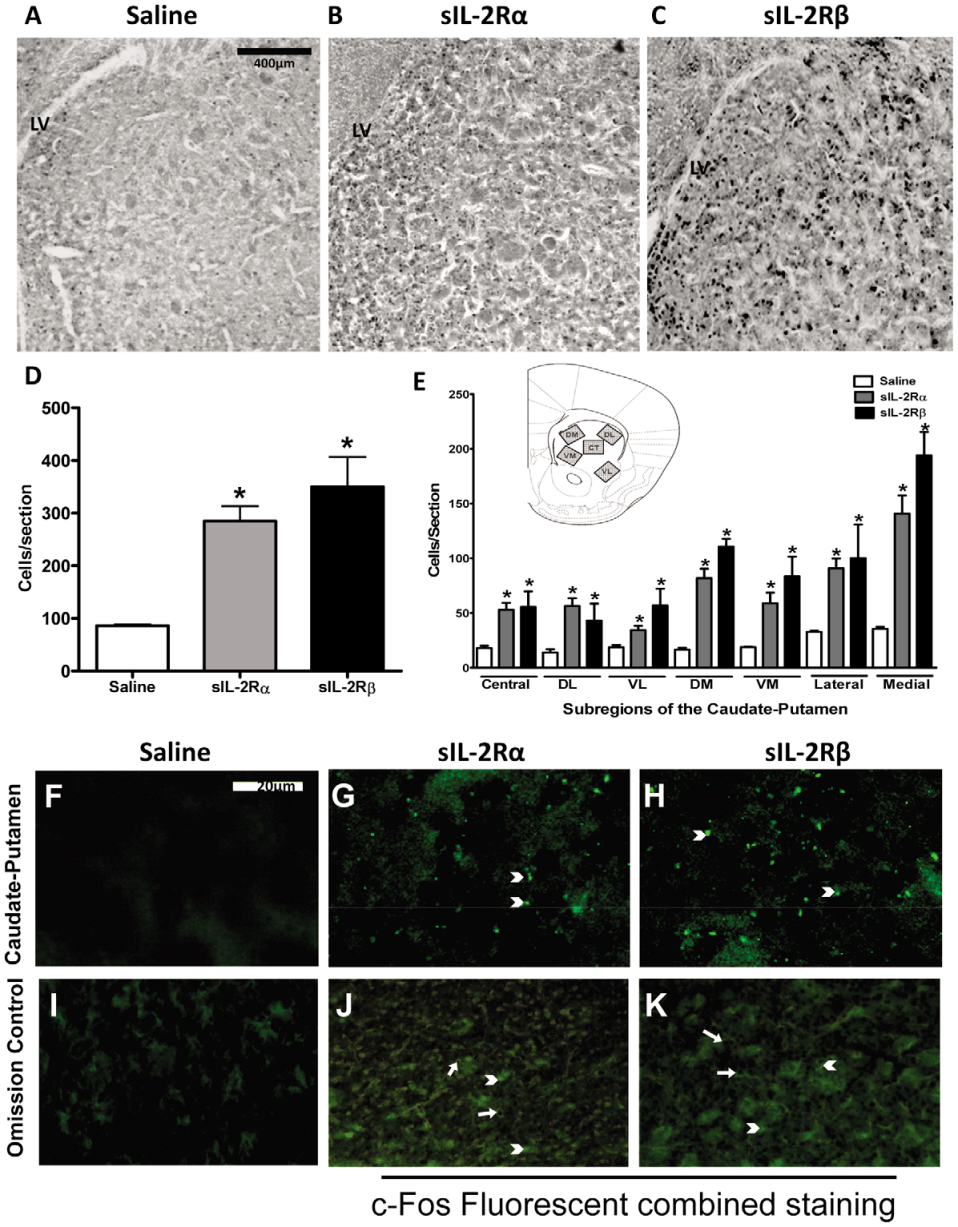
c-Fos expression and Fluorescent staining of Caudate Putamen in sIL-2Rs treated mice. Photomicrographs of brain sections showing Fos-like immunoreactive cells in the caudate–putamen of mice receiving single injections of (A) saline, (B) sIL-2Rα (1 µg, s.c.), and (C) sIL-2Rβ (2 µg, s.c.). (D) Histogram showing the number (mean ± S.E.M.) of Fos-positive cells in the caudate–putamen after administration of saline or single injections of sIL-2Rα or sIL-2Rβ. (E) Histogram showing the number (mean ± S.E.M.) of Fos-positive cells counted within the indicated subterritories of caudate–putamen after administration of saline or single injections of sIL-2Rα or sIL-2Rβ (*n* = 4). Insert reflects regions sampled for counting Fos-positive cells in the dorsomedial (DM), dorsolateral (DL), central (CT), ventrolateral (VL) and ventromedial (VM) aspects of the caudate–putamen. Photomicrographs showing deposits of sIL-2Rα or sIL-2Rβ in caudate–putamen after administration of (F) saline or single injections of (G) sIL-2Rα and (H) sIL-2Rβ. Photomicrograph of omission control (I). Photomicrographs showing merged c-Fos and Fluorescent staining in sIL-2Rα treated mice (J) and sIL-2Rβ treated mice (K). [Arrow head: - Fluorescent staining; Arrow: - c-fos staining].

A marked increase in c-Fos expression was induced by sIL-2Rs administration in the caudate-putamen, F (2, 27) = 7.530, p = 0.00007 (figures 3A–3E). Student-Newman-Keuls multiple comparisons (α = 0.05) confirmed that compared to controls, Fos-like immunoreactivity was significantly elevated in sIL-2Rα-treated mice (by 1.85 fold) and sIL-2Rβ-treated mice (by 7 fold). Post-hoc analyses further revealed that c-Fos expression of sIL-2Rβ-treated mice significantly exceeded those of mice receiving sIL-2Rα. It is also important to note that a heterogeneous pattern of c-Fos immunoreactivity was induced by both sIL-2R subunits. Indeed, regional analyses indicated that compared with controls, Fos expression was significantly elevated within the dorsomedial, ventromedial, dorsolateral, ventrolateral, and central caudate in sIL-2Rα- and sIL-2Rβ-treated mice, and that the magnitude of these increases were significantly greater in mice receiving sIL-2Rβ.

**Figure 4:**
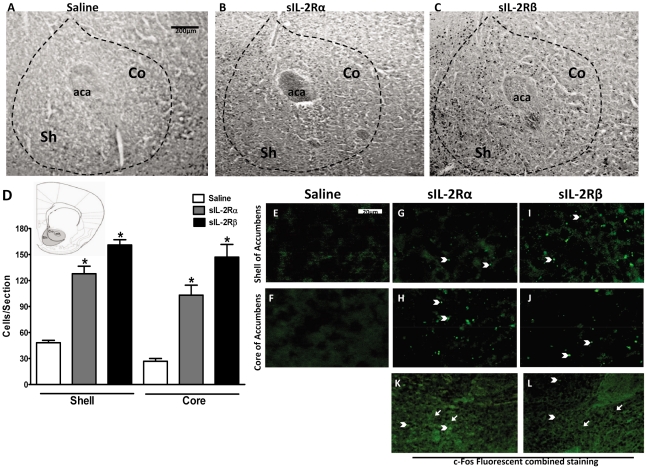
c-Fos expression and Fluorescent staining of Nucleus Accumbens in sIL-2Rs treated mice. Photomicrographs of brain sections showing Fos-like immunoreactive cells in the nucleus accumbens of mice receiving single injection of (A) saline, (B) 1 µg sIL-2Rα or (C) 2 µg sIL-2Rβ. Abbreviations: aca, anterior commissure; Sh, shell of nucleus accumbens; Co, core of nucleus accumbens. (D) Histogram show the number (mean ± S.E.M.) indicate Fos-positive cells counted within the indicated compartments of the nucleus accumbens after administration of saline or single injections of sIL-2Rα or sIL-2Rβ. Photomicrographs of deposits of sIL-2Rα or sIL-2Rβ in shell or core of nucleus accumbens after administration of (E and F) saline or single injections of (G and H) sIL-2Rα or (I and J) sIL-2Rβ. Photomicrographs showing merged c-Fos and Fluorescent staining in sIL-2Rα treated mice (K) and sIL-2Rβ treated mice (L). [Arrow head: - Fluorescent staining; Arrow: - c-fos staining].

sIL-2Rs treatment induced profound increases in Fos expression in the nucleus accumbens, F(2,25) = 4.346, p = 0.0250 (figures 4A–D). Student Newman-Keuls multiple comparisons confirmed that the total number of Fos-like immunoreactive cells in the accumbens was significantly elevated in sIL-2Rα treated (by 28 fold) mice and in sIL-2Rβ treated mice (by 24 fold) compared to saline-treated mice (figure 4D). Although both soluble receptor subunits induced increases in Fos expression that were similar in magnitude, there were striking differences in the regional distribution of these effects. Irrespective of sIL-2R subtype, sIL-2R induced Fos expression was significantly greater in the shell of the accumbens than in the core [F (5, 31) = 3.912, p = 0.0288; figure 4D]. Moreover, throughout the accumbens, Fos expression in sIL-2Rβ treated animals was found more profound than sIL-2Rα [F (5, 31) = 3.671, p = 0.0315; figure 4D].

**Figure 5:**
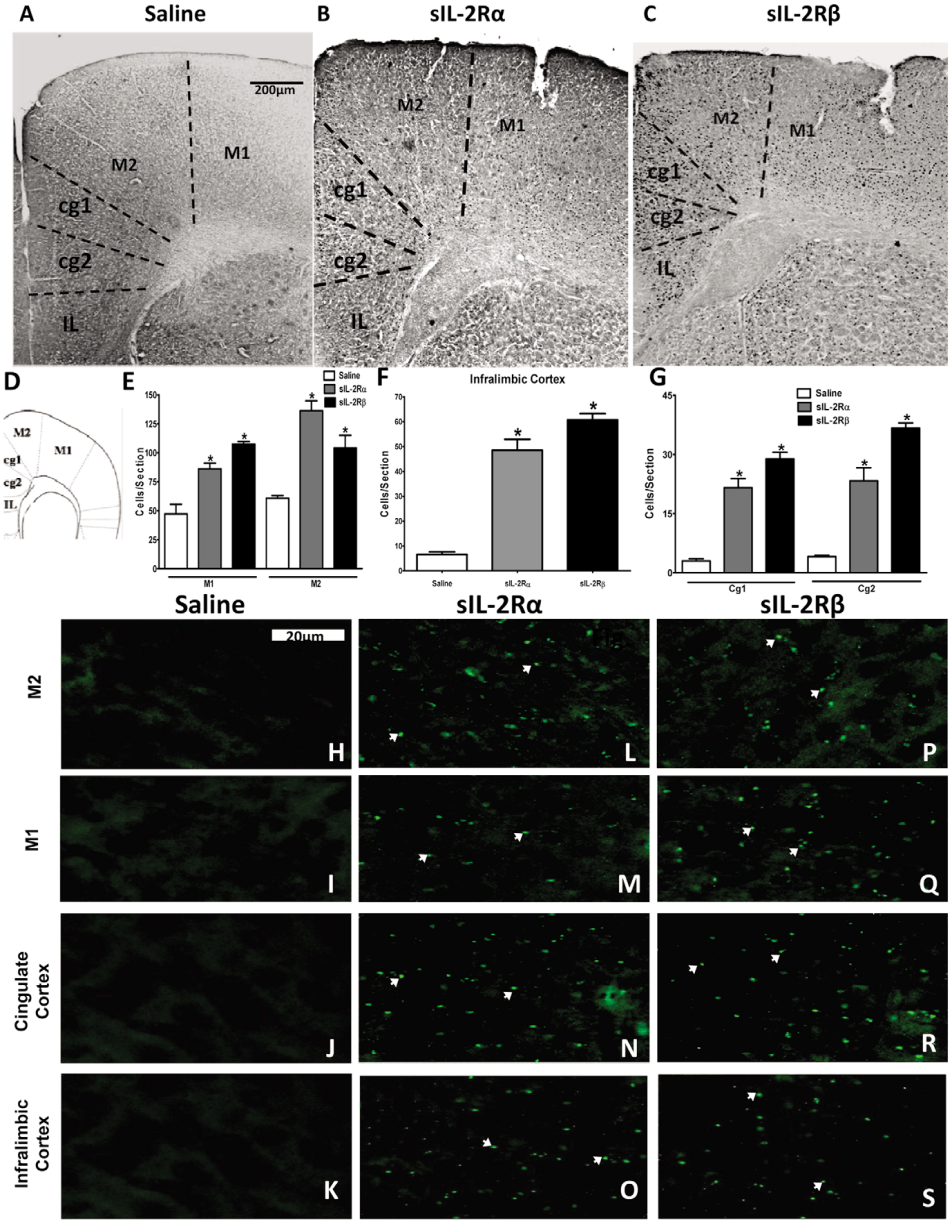
c-Fos expression and Fluorescent staining of Cortices in sIL-2Rs treated mice. Photomicrographs of brain sections showing Fos-like immunoreactive cells in the motor cortex, cingulate cortex and infralimbic cortex of mice receiving single injection of (A) saline, (B) 1 µg sIL-2Rα or (C) 2 µg sIL-2Rβ. (D) Insert reflects regions sampled for counting Fos-positive cells in the motor cortex, cingulate cortex and infralimbic cortex. Histogram shows the number (mean ± S.E.M.) of Fos-positive cells counted within the indicated compartments of the (E) motor cortex, (F) cingulate cortex and (G) infralimbic cortex after administration of saline or single injections of sIL-2Rα or sIL-2Rβ. Photomicrographs of deposits of sIL-2Rα or sIL-2Rβ in (H, L and P) M1 division of motor cortex, (I, M and Q) M2 division of motor cortex, (J, N and R) cingulate cortex, and (K, O and S) infralimbic cortex.

Administration of both sIL-2Rs increased Fos-like immunoreactivity within motor cortex [F (5, 35) = 27.22, p = 0.0036; figure 5A]. In sIL-2Rα-treated mice, Fos expression was increased predominantly in the M2 part of motor cortex, which is responsible for planning and coordination of complex movements (figures 5B and 5E). Likewise, both sIL-2Rs increased Fos expression in infralimbic and cingulate cortices (figures 5C, 5F and 5G), which are believed to be linked more closely with emotional behaviors. However, greater quantities of labeling were manifest in these regions following sIL-2Rβ administration.

**Figure 6:**
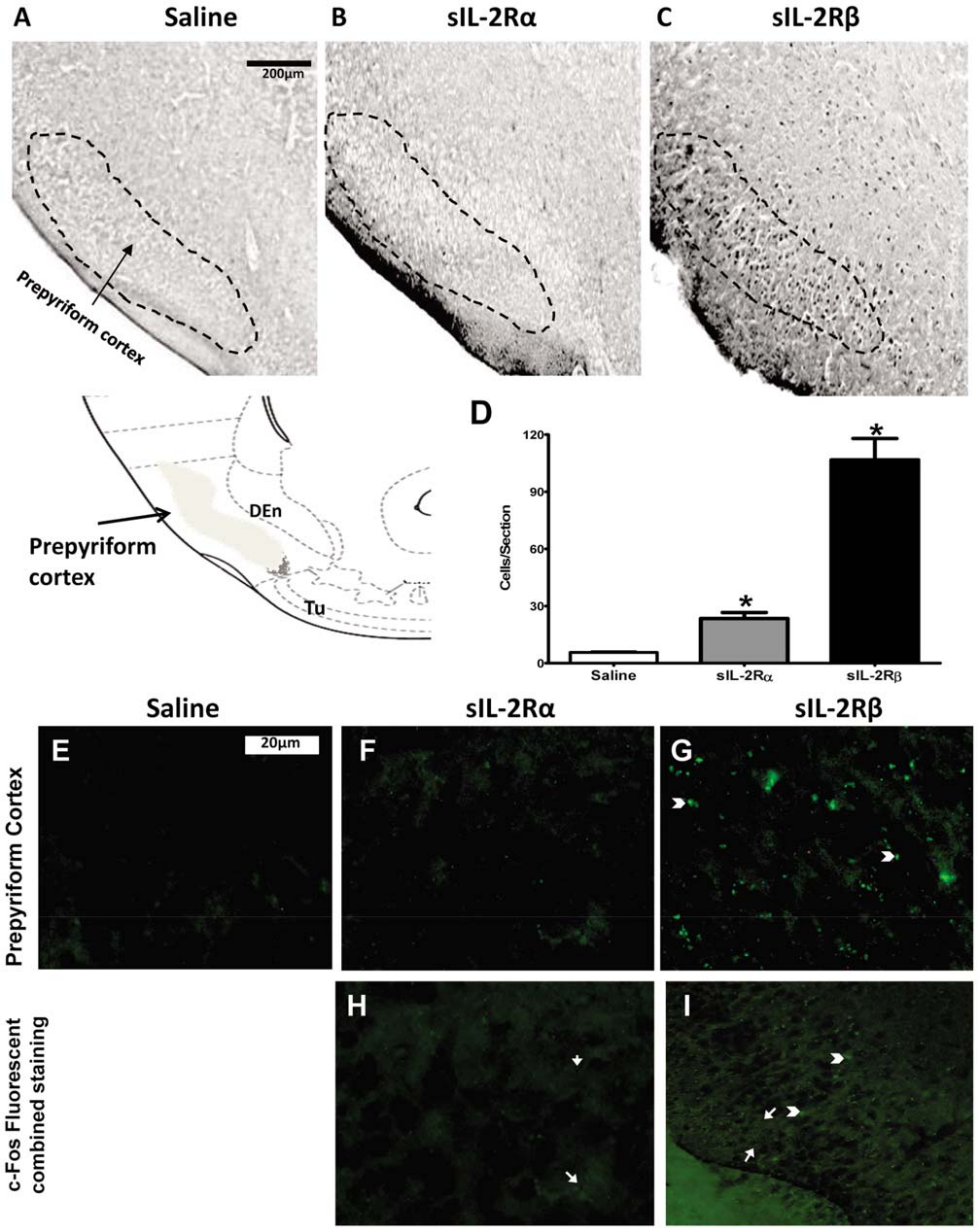
c-Fos expression and Fluorescent staining of Prepyriform Cortex in sIL-2Rs treated mice. Photomicrographs of brain sections showing Fos-like immunoreactive cells in the prepyriform cortex of mice receiving single injection of (A) saline, (B) 1 µg sIL-2Rα or (C) 2 µg sIL-2Rβ. (D) Number (mean ± S.E.M.) of Fos-positive cells in the prepyriform cortex after administration of saline or single injections of sIL-2Rα or sIL-2Rβ. Photomicrographs of deposits of sIL-2Rα or sIL-2Rβ in prepyriform cortex after administration of (E) saline or single injections of (F) sIL-2Rα or (G) sIL-2Rβ. Photomicrographs showing merged c-Fos and Fluorescent staining in sIL-2Rα treated mice (H) and sIL-2Rβ treated mice (I). [Arrow head: - Fluorescent staining; Arrow: - c-fos staining].

In prepyriform cortex, which is believed to be an extension of primary olfactory cortex, significant 5 fold increases in induction of Fos-like immunoreactivity were only shown in sIL-2Rβ treated mice, as shown in figures 6A–6D [F (2, 17) = 18.47, p = 0.0004].

### Recombinant sIL-2Rα and sIL-2Rβ accumulation in the brain

The present findings show for the first time that a soluble receptor modulates neural activity. How they affect brain activity is not known. We thus sought to determine whether these receptors are localized within the brain. As illustrated, recombinant sIL-2Rα and sIL-2Rβ deposits were found at the level of the rostral caudate nucleus (figures 3F–3I), shell and core of nucleus accumbens (figures 4E–4J), and motor cortex (figures 5H–5S). In sIL-2Rα treated mice, the deposits of recombinant sIL-2Rα were more prominent in the rostral caudate nucleus, M2 part of motor cortex and core of nucleus accumbens; and for the recombinant sIL-2Rβ treated mice, the deposits of recombinant sIL-2Rβ were also more prominent in the rostral caudate nucleus, M2 component of motor cortex, and in the shell of the nucleus accumbens. The deposits in prepyriform cortex were found only in sIL-2Rβ treated mice but not in sIL-2Rα treated mice (figures 6E–6G). The label reflected injected and not endogenous sIL-2Rs since mice received human sIL-2Rs.

We also sought to determine the extent to which there exists an overlap of label of c-fos and sIL-2Rα in the forebrain regions in question by overlying the photomicrographs for each label from the same regions, one upon the other (in the absence of performing double labeling procedures which were not conducted because of incompatibilities between the staining procedures for these methods). It was observed that there was significant overlap in staining for c-Fos and sIL-2Rs in different regions of the caudate nucleus. Specifically, as shown in Fig. 3 (J–K), extensive overlap was observed between sIL-2Rs and c-fos. Similar findings were also observed for the nucleus accumbens (Fig. 4, K–L). However, as there was no fluorescent labeling in the prepyriform cortex for sIL-2Rα treated mice, combining c-fos and fluorescent labeling only indicated the presence of c-fos expression in this region as compared to the overlapping of both types of label in sIL-2Rβ treated mice in this region (Fig. 6 H–I).

## Discussion

The major discovery derived from the present study was that our findings demonstrated that administration of soluble cytokine receptors led to disturbances in motor behavior. This conclusion is evident from two forms of observations described in the Results Section. These include: (1) the behavioral manifestations of soluble IL-2 receptor administration and (2) their presence in specific regions of the brain related to motor functions. With respect to the first set of observations, we discovered that single injections of sIL-2Rα or sIL-2Rβ (0–2 µg/Balb/c mouse; s.c.) induce marked subunit-specific alterations in novelty-induced locomotion and repetitive stereotypic behaviors. Specifically, sIL-2Rα increased the incidence of in place stereotypic motor behaviors, including rearing, turning, and grooming behavior. A wider spectrum of behavioral changes was evident in mice treated with sIL-2Rβ, including increases in vertical and horizontal ambulatory activity and stereotypic behaviors. In contrast with sIL-2Rs, soluble IL-1 Type 1 receptors (1–8 µg, sc) did not affect novelty-induced behavioral motor responses, providing further support for the specificity of sIL-2R-induced behavioral variations. Concerning the second aspect of our findings, it was observed that there was a general overlap of c-Fos expression and soluble IL-2 receptor labeling within regions of the forebrain, which include the caudate nucleus, motor cortex, nucleus accumbens, and prepyriform region that have been traditionally associated with the behavioral motor processes described in the present study. sIL-2Rs induced pronounced subunit-specific alterations of Fos expression in the striatum and cortex. We also discovered that sIL-2Rα and sIL-2Rβ localized in the cortex and striatum and that there was specificity with regard to this localization. Indeed, there is a striking similarity in the distributions of Fos-like immunoreactivity and presence of sIL-2Rs, suggesting that they induce local effects in these brain regions.

Based on c-Fos data, it can be presumed that the caudate, nucleus accumbens and motor cortex are associated with exploratory behavior and with in-place stereotypic activities in a novel environment, whereas, the prepyriform cortex is associated with enhanced locomotor activity. Additionally, higher c-fos activation in the medial aspect of the caudate nucleus relative to the lateral aspect in sIL-2Rβ treated mice suggests involvement of this region in locomotor and stereotypic behaviors. Moreover, c-Fos activation was observed in the M2 region of motor cortex of sIL-2Rα treated mice coupled with higher levels of activation in the M1 region of motor cortex in sIL-2Rβ treated mice, suggesting a possible role of M2 in fine motor (in-place) activities and M1 in gross motor (exploratory locomotor) activities.

From the present findings, it is now clear that in addition to acting in antagonistic manners, soluble cytokine receptors, including sIL-2Rs, may potentiate lymphoid and cytokine activity in normal and disease states [3]. The IL-2 receptor is normally expressed on several cell types, including T cells, B cells, natural killer cells, and monocytes [4]. It is composed of α, β and γ subunits. The α subunit confers individuality to IL-2, while the β and γ subunits are shared with other cytokines (notably IL-15) [22]. After being shed from activated T and B cells and released into the circulation, sIL-2Rs bind IL-2. Increases in sIL-2R are evident in various disorders involving T cell activation, including autoimmune and neoplastic diseases. Thus, it has been suggested that circulating levels of sIL-2R may be used as diagnostic markers, and perhaps as predictors of disease activity. [7], [23]

In recent years, it has become apparent that soluble cytokine receptors have additional biological functions. For example, the soluble TNFR1 helps modulate the transport of substances across the blood brain barrier [24]. Such findings raise the possibility that a soluble cytokine receptor may affect brain function, and thus, play a role in the disease process. Circulating soluble cytokine receptors, such as soluble interleukin-2 receptors (sIL-2R) are normal constituents of body fluids that regulate peripheral cytokine and lymphoid activity. Peripheral sIL-2Rs are elevated in psychiatric and neurological disorders linked with autoimmune processes [7]–[8], including ones involving an increased expression of repetitive stereotypic behaviors [11]. However, there is no evidence that sIL-2Rs (or any soluble receptor) modulate such behavior or that they localize in the brain.

The question may be raised of whether or not there exists a relationship between specific levels of endogenous IL-2 in regions of the forebrain related to motor functions considered in this study and levels of sIL-2R and metabolic levels of brain activity for these regions as implied from c-fos analyses. The presence of such a relationship would provide further significance to the potential functional importance of sIL-2R in the regulation of motor behaviors related to various disease states related to movement disorders. Unfortunately, at the present time, such information appears to be lacking in the literature. Therefore, one natural extension of the present study would be an attempt to provide an analysis of the relationship characterizing c-fos levels associated with specific motor responses as well as an attempt to identify the possible relationship between sIL-2R with endogenous levels of IL2 for specific regions of the forebrain related to the motor responses considered in this paper.

In summary, we show for the first time that: (1) soluble receptors (sIL-2R) induce behavioral changes, which include stimulation of locomotion and repetitive stereotypic behaviors; (2) these effects are (a) subunit-specific and (b) cytokine receptor-specific. We also discovered that soluble receptors induce subunit-specific variations of cell activity in the striatum and cortex. A striking finding is that sIL-2Rs localized in these regions suggesting that sIL-2Rs locally modulate brain function. These findings imply that soluble cytokine receptors are novel modulators of brain function and act as immune-brain messengers, which may be helpful in the future for identifying potential etiological agents and possible therapeutic targets.
